# American Academy of Dental Science

**Published:** 1885-11

**Authors:** 


ARTICLE VIII.

### AMERICAN ACADEMY OF DENTAL SCIENCE.

At the 18th Annual Meeting of the American Academy of Dental Science, held in Boston, November 4th, 1885, the address was delivered by Dr. W. C. Barrett, of Buffalo. Subject.—"The Diseases of the Period of Dentition."

The following officers were chosen for the ensuing year.

*President.* Dr. J. H. Batchelder.

*Vice-President.* Dr. C. P. Wilson.

*Rec. Secretary.* Dr. E. E. Hopkins.

*Cor. Secretary.* Dr. E. B. Hitchcock.

*Treasurer.* Dr. E. H. Smith.

*Librarian.* Dr. H. C. Meriam.

*Executive Committee:* Drs. Chas. Wilson, E. C. Briggs, J. S. Mason.

It was resolved that the American Academy of Dental Science learns with gratification of the re-establishment of a section on Dental and Oral Surgery in the proposed Medical Congress of 1887.

Boston, November 5th. E. E. HOPKINS, *Secretary.*

